# An excitatory ventral hippocampus to lateral septum circuit that suppresses feeding

**DOI:** 10.1038/ncomms10188

**Published:** 2015-12-15

**Authors:** Patrick Sweeney, Yunlei Yang

**Affiliations:** 1 Department of Neuroscience and Physiology, State University of New York Upstate Medical University, 505 Irving Avenue, IHP#3609, Syracuse, New York 13210, USA

## Abstract

Previous research has focused on feeding circuits residing in the hindbrain and midbrain that govern homeostatic or hedonic control of food intake. However, the feeding circuits controlling emotional or cognitive aspects of food intake are largely unknown. Here we use chemical genetics and optogenetic techniques to dissect appetite control circuits originating from ventral hippocampus (vHPC), a brain region implicated in emotion and cognition. We find that the vHPC projects functional glutamatergic synaptic inputs to the lateral septum (LS) and optogenetic activation of vHPC projections in LS reduces food intake. Consistently, food intake is suppressed by chemogenetic activation of glutamatergic neurons in the vHPC that project to the LS and inactivation of LS neurons blunts vHPC-induced suppression of feeding. Collectively, our results identify an anorexigenic neural circuit originating from vHPC to LS in the brain, revealing a potential therapeutic target for the treatment of anorexia or other appetite disorders.

## Abstract (web summary)

The ventral hippocampus connects to the hypothalamus and has been implicated in feeding behaviours. Here, the authors use a combination of optogenetics and DREADD strategies to dissect the underlying circuit, showing that projections from the vHC to the lateral septum work to regulate feeding suppression.

## Graphical abstract

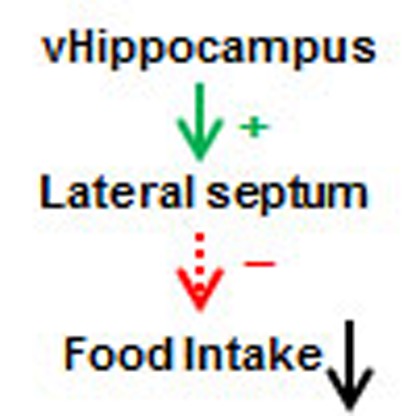

Much effort has been focused on understanding hypothalamic regulation of feeding behaviour^1^,^2^,^3^ and the functions of the hippocampus in memory and anxiety^4^,^5^,^6^,^7^,^8^,^9^. Hypothalamic brain regions are inter-connected in neural circuits within the limbic system that includes hippocampus. For example, the ventral hippocampus (vHPC), a medial temporal lobe structure with a prominent role in cognitive and emotional behaviours^7^,^8^,^9^, is directly linked to rostral hypothalamus^10^. In addition to the involvement of vHPC in cognitive and emotional processes, several lines of evidence demonstrate that the vHPC also contributes to the regulations of feeding and energy homeostasis^11^,^12^. For instance, selective neurotoxic lesions of the vHPC promote food intake and induce body weight gain in rodents^13^,^14^,^15^. Strikingly, it is well-demonstrated that the receptors for the anorexigenic hormones leptin and insulin and the orexigenic hormone ghrelin are expressed in the vHPC in addition to the well-known locations in the hypothalamus and hindbrain^16^,^17^,^18^. Moreover, recent studies have demonstrated that feeding behaviour is negatively regulated by hippocampal leptin and glucagon-like peptide-1 signalling^19^,^20^. By contrast, administration of ghrelin within the vHPC increases feeding^21^. Collectively, these findings indicate that the vHPC is capable of regulating food intake in a bidirectional manner, although the underlying neural circuits remain unknown. We thus proposed that different neuron populations in the vHPC may exert distinct functions in the regulation of feeding, analogous to the distinct functions of orexigenic agouti-related protein and anorexigenic pro-opiomelanocortin neurons in the hypothalamic arcuate nucleus^2^.

In this study, we focused on identifying the neuron populations in the vHPC that are involved in vHPC regulation of food intake and deciphering the underlying neural circuits. By using approaches that include retrograde tracing, Cre-Lox technology, chemogenetics, optogenetics and Channelrhodopsin-2 (ChR2)-assisted circuit mapping, we find that food intake is reduced by selective chemogenetic activation of vHPC glutamatergic neurons projecting to the LS. Consistently, food intake is facilitated by inactivation of vHPC neurons projecting to the LS. Moreover, chemogenetic inactivation of the LS neurons blocks vHPC-induced suppression of appetite. Taken together, these results indicate that vHPC exerts anorexigenic effects on food intake by projecting glutamatergic synaptic inputs to the LS, although we cannot exclude other signalling pathways.

## Results

### Activation of vHPC reduces feeding

**Figure 1:**
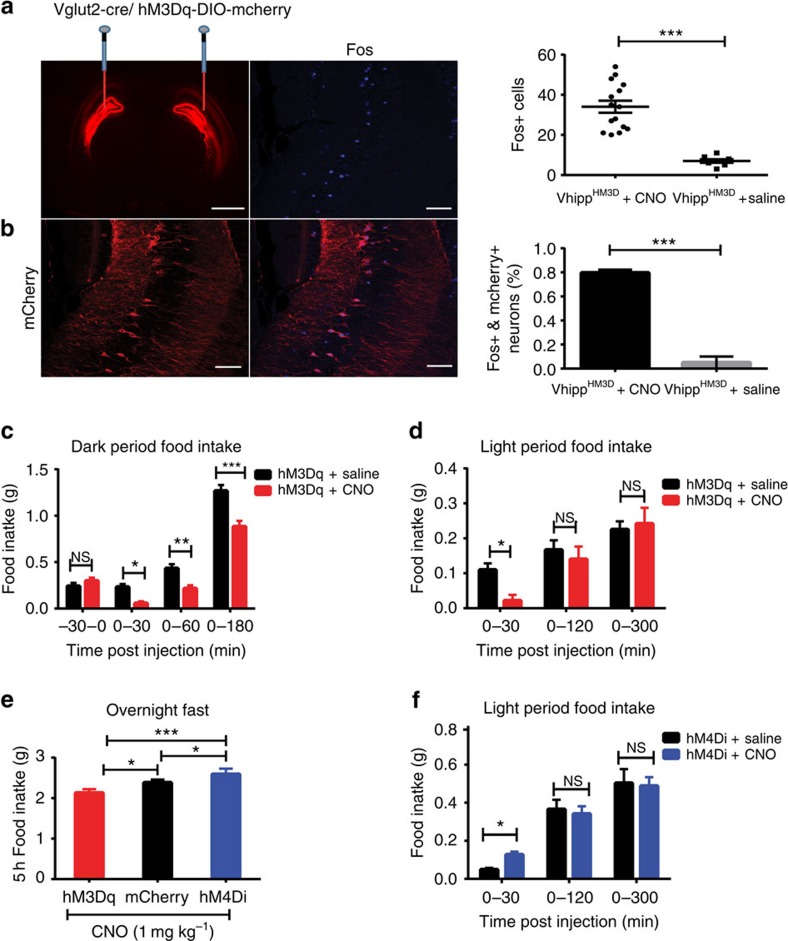
Chemical genetic activation of vHPC glutamatergic neurons suppresses food intake. (**a**) Representative images showing bilateral expression of hM3Dq-mCherry (left panel), Fos expression in vHPC DG–CA3 neurons (middle panel) and quantification (right panel) of Fos in the ventral hippocampus of Vglut2-Cre mice. CNO (1 mg kg^−1^; i.p.) or saline injections were administered 30 mins prior to perfusion. Data points represent Fos quantification in individual brain slices (*n*=15 slices per 3 mice for CNO treatment; *n*=8 slices per 2 mice for saline treatment; unpaired Student's *t*-test, *t*=6.424; df=21, *P*<0.001). (**b**) hM3Dq-mCherry transfection in CA3 region (left panel) with overlapping Fos positive neurons (middle panel) and a quantification of the percentage of mCherry-positive neurons co-expressing Fos (right panel; *n*=2–3 mice per group; unpaired Student's *t*-test, *t*=14.44; df=17, *P*<0.001). (**c**,**d**) Feeding assays showing that chemical genetic activation of vHPC DG–CA3 neurons reduced food intake during both the dark period (**c**; *n*=6 mice; F_2,44_=7.417, *P*=0.0017) and light period (**d**; *n*=6 mice; two-way ANOVA with Sidak's *post hoc* test; F_1,68_=5.692, *P*=0.0198) of the rodent light cycle. (**e**) Following an overnight fast, activation of vHPC DG–CA3 neurons reduced food intake while inhibition of DG–CA3 neurons increased food intake (*n*=5 mice for mCherry group; *n*=5 mice for hM3Dq group and *n*=6 for hM4Di group; two-way repeated measures ANOVA with Holm-Sidak's *post hoc* test; F_2,29_=7.311, *P*=0.003). (**f**) Light period feeding assays indicating that food intake was increased 30 mins following chemical genetic inhibition of vHPC DG–CA3 glutamate neurons (*n*=6 mice; unpaired Student's *t*-test, *t*=4.83; df*=*28, *P*<0.05). Data represent mean±s.e.m. unless otherwise noted. All behaviour experiments and treatment conditions were repeated at least two times per animal; *P<0.05, **P<0.01, ***P<0.001; FI (food intake); Scale bars, 1 mm for 1**a** (left panel); and 50 μm for all other figures. ANOVA, analysis of variance; NS, not significant.

Several lines of evidence demonstrate that vHPC modulates food intake^11^,^12^,^13^,^14^,^15^,^19^,^20^,^21^. However, the neuron populations and underlying neural circuits involved in vHPC control of feeding remain unknown. To identify the neuron populations and the neural circuits, a chemogenetic DREADD (Designer Receptors Exclusively Activated by Designer Drugs) approach was used in combination with feeding assays. DREADD technology uses engineered G protein-coupled receptors (GPCRs) that are exclusively activated by synthetic ligands. For instance, the engineered human muscarinic type 3 GPCR (hM3Dq) is unresponsive to all known endogenous ligands. Instead, hM3Dq can be activated by the otherwise pharmacologically inert compound clozapine-N-oxide (CNO)^22^,^23^. It is well-demonstrated that glutamatergic neurons are abundant within the vHPC and are involved in hippocampus-mediated behaviours ranging from addiction to anxiety and memory^24^,^25^,^26^. To investigate the potential roles of these neurons in feeding behaviour, we took advantage of viral delivery approaches, as detailed in the previous study^27^ and in the Methods. Bilateral injections of Cre-dependent viral vectors expressing hM3Dq-mCherry were performed in Vglut2-Cre transgenic mice to transduce hM3Dq-mCherry in the neurons within the dentate gyrus (DG) and CA3 subregions of the vHPC (Fig. 1a). This area was targeted since it is reported that these regions are the exclusive hippocampal locations for anorexigenic leptin receptors^26^, suggesting that activation of the glutamatergic neurons localized in the ventral hippocampal DG or CA3 subregions may exert inhibitory effects on feeding behaviour. We thereafter designated these neurons as ventral hippocampal DG–CA3 (vhDG–CA3) glutamatergic neurons.

It has been well-demonstrated that the stimulatory DREADD-hM3Dq couples through G_q_ pathways to depolarize neurons^22^,^23^. DREADD-based *in vivo* activation of vhDG–CA3 neurons was achieved by intraperitoneal (i.p.) injections of CNO (1 mg kg^−1^ in 200 μl saline), as indicated by increased Fos expression in response to i.p. CNO injections (Fig. 1a,b). Next, we tested the ability of vhDG–CA3 glutamatergic neurons to regulate food intake. We found that administration of CNO suppressed food intake during both the light and dark periods of the rodent light cycle (Fig. 1c,d) when compared with control mCherry-transduced mice (Supplementary Fig. 1). Consistently, refeeding after overnight food deprivation was also suppressed following activation of vhDG–CA3 neurons (Fig. 1e). Food intake was significantly reduced for 3 h or 5 h, respectively, during evening or overnight fasting experiments. During the daytime, when mice usually consume little, we noticed that activation of vhDG–CA3 neurons produced a transient decrease in food intake which lasts about 30 mins after CNO injections. Consistently, repeated dosing of CNO with twice daily injections to hM3Dq-transduced mice did not exert significant effects on daily food intake (Supplementary Fig. 2). The transient changes in feeding during daytime hours are likely compensated by the predominant feeding circuits localized in hypothalamic brain regions, supported by the highly redundant, multiplexed mechanisms and neural circuits that govern feeding behaviour^1^,^2^,^3^. Further studies are needed to investigate the relationships of the neural circuitry underlying ventral hippocampal and hypothalamic regulation of food intake.

### Inactivation of vHPC facilitates feeding

To exclude the possibility that DREADD-based ventral hippocampal suppression of food intake is secondary to changes in behaviours that are incompatible with feeding, we performed reverse experiments with bilaterally transducing the inhibitory DREADD-hM4Di into vhDG–CA3 glutamatergic neurons in Vglut2-Cre mice. It is well-demonstrated that, in contrast to DREADD-hM3Dq, the inhibitory DREADD-hM4Di couples through G_i_ signalling pathways to inhibit neuronal activity and synapse transmission in response to CNO stimulation^22^,^23^,^28^. As expected, contrasting to the reduction of food intake by DREADD-hM3Dq activation of vhDG–CA3 neurons (Fig. 1c-e), we observed that food intake was increased in response to CNO administration (Fig. 1f) in mice transduced with hM4Di. Consistently, we also observed that DREADD-mediated inhibition of vhDG–CA3 neurons significantly increased refeeding after food deprivation in comparison to mCherry- and hM3Dq-transduced mice (Fig. 1e).

**Figure 2:**
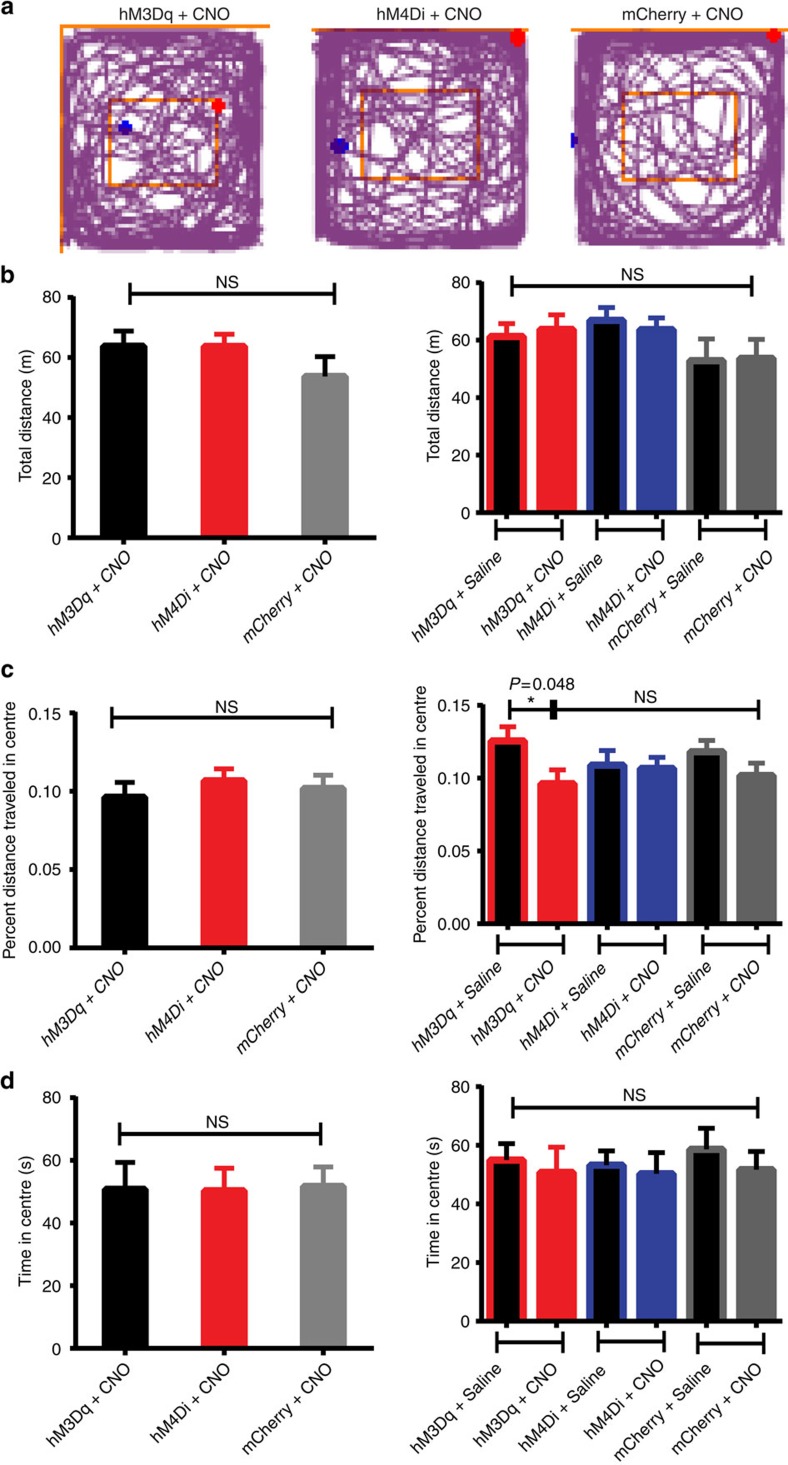
Activation or inactivation of vHPC glutamatergic neurons has minimal effects on locomotion and anxiety. The Vglut2-Cre mice transduced with the stimulatory hM3Dq (*n*=5), the inhibitory hM4Di (*n*=6) or control mCherry (*n*=5) in vhDG–CA3 glutamatergic neurons were monitored for 10 mins during open field exploration to assay for changes in anxiety or locomotor activity in response to CNO (1 mg kg^−1^) or saline injections. (**a**) Representative sample traces showing 10 mins of open field exploration following i.p. injections of CNO (1 mg kg^−1^) for mice transduced with hM3Dq (left panel), hM4Di (middle panel) or mCherry (right panel). Blue dots indicate the mouse's staring point and red dots indicate the final position of the mouse. (**b**) Total distance travelled during 10 mins of open field exploration. No significant differences were detected in the transduced mice with CNO or saline injections in each group (left panel: F_2,24_=0.93, *P*=0.41; right panel: F_5,47_=0.99, *P*=0.43; one-way ANOVA). (**c**) Quantification of the percent distance travelled in the centre of the open field. No significant differences were observed between animals treated with CNO (left panel: F_2,24_=0.42, *P*=0.66; one-way ANOVA). A slight anxiogenic effect was detected in percent distance travelled in the centre of the open field when comparing hM3Dq-transduced mice with vehicle saline conditions (right panel; unpaired Student's *t*-test, *t*=2.14, df=18.0, *P*=0.05). All other comparisons were statistically insignificant (right panel; F_5,47_=0.99, *P*=0.43; one-way ANOVA). (**d**) Time spent in the centre of the open field during 10 mins of open field exploration. No significant differences were detected in the transduced mice with CNO or saline injections in each group (left panel: F_2,24_=0.009, *P*=0.99; right panel: F_5,46_=0.21, *P*=0.96; one-way ANOVA). I.p. injections of CNO (1 mg kg^−1^) and saline were administered on consecutive days 10 mins prior to placing animals in the open field. Open field experiments were repeated 2 weeks later in the opposite order and average values were calculated for each mouse. Data represent mean±s.e.m. ANOVA, analysis of variance; *P<0.05; NS, not significant.

We next tested if DREADD-based activation or inactivation of vhDG–CA3 neurons affected physical activity or anxiety. We performed open field behavioural testing in Vglut2-Cre mice transduced with DREADD-hM3Dq, DREADD-hM4Di or control mCherry in vhDG–CA3 glutamatergic neurons as described above for feeding assays. We observed that DREADD-based activation of vhDG–CA3 neurons produced no noticeable changes in locomotion following both 10 mins and 30 mins of open field exploration (Fig. 2a,b; Supplementary Fig. 3). In addition, minimal changes in anxiety were observed following activation of vhDG–CA3 neurons as both time spent in the centre and percent distance travelled in the centre of the open field did not show apparent changes with CNO treatments in comparison to DREADD-hM4Di and mCherry-transduced mice (Fig. 2c,d). However, we observed that CNO activation of vhDG–CA3 neurons reduced percent distance travelled in the centre relative to vehicle saline conditions (Fig. 2c; right panel), suggesting a potential anxiogenic effect following activation of vhDG–CA3 neurons. This apparent anxiogenic effect is minimal and does not interfere with the ability of animals to seek food since locomotion and exploration of the animals are not affected (Fig. 2a–d; Supplementary Fig. 3). Collectively, these results indicate that vHPC participates in the control of food intake through underlying neural circuits which we further investigate as follows.

### Activation of LS projections of vHPC neurons reduces feeding

**Figure 3:**
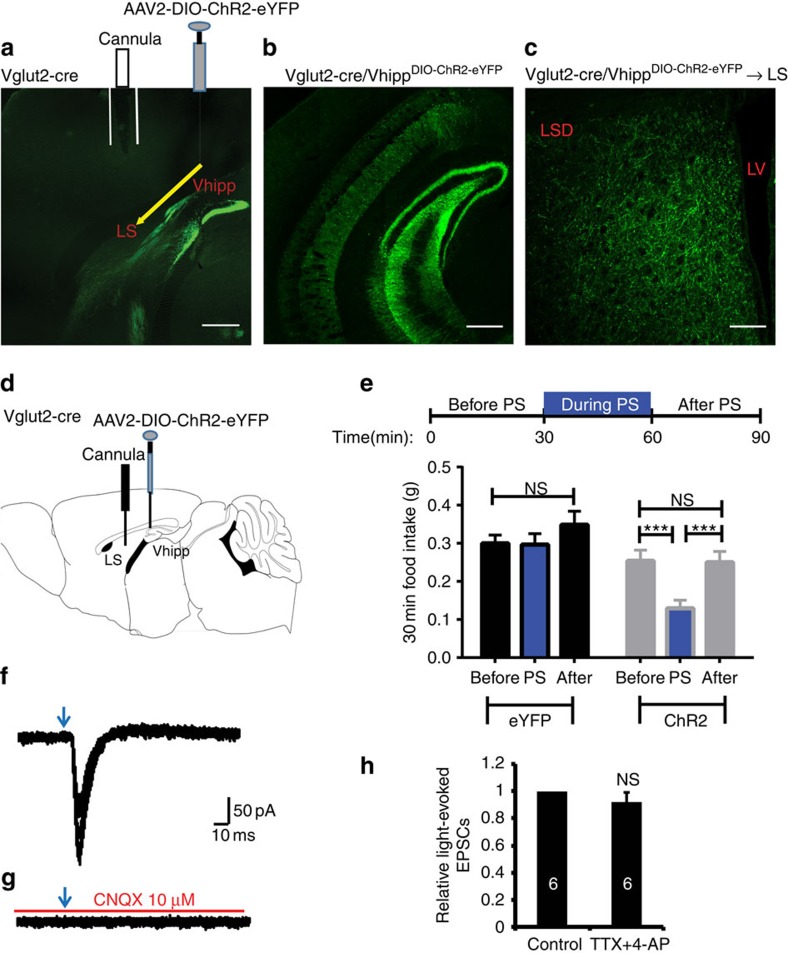
Optogenetic dissection of the vHPC to LS neural circuit that suppresses food intake. (**a**) Representative sagittal section showing Cre-dependent ChR2 expression in the ventral hippocampus in Vglut2-Cre mouse with cannula placement above the LS. (**b**,**c**) Coronal images of ChR2 expression in the ventral hippocampus (**b**) corresponding projection fibres in the LS (**c**). (**d**) Schematic illustration of viral injections and cannula placement for optogenetic stimulation. (**e**) Outline of optogenetic experiments (top panel) and behavioural data (bottom panel) showing that *in vivo* photostimulation of vHPC→LS glutamatergic fibres suppressed food intake (*n*=8 and 5 mice for ChR2 and eYFP control groups, respectively; F_2,54_=3.985, *P*=0.0243; two-way ANOVA with Tukey's *post hoc* test). (**f** and **g**) ChR2-assisted circuit mapping indicates functional glutamatergic projections from the ventral hippocampus to the lateral septum, as indicated by light-evoked synaptic currents that were completely eliminated in the presence of the AMPA receptor blocker CNQX (*n*=11). (**h**) The amplitudes of light-evoked synaptic currents were not significantly affected with the treatment of TTX and 4-AP (*n*=6 neurons per 6 slices per 2 mice; paired student's *t*-test). Results represent mean±s.e.m.; all behavioural experiments and treatment conditions were repeated at least two times per animal; ***P<0.001. Scale bars, 500 μm for **a**,**b** and 150 μm for **c**. ANOVA, analysis of variance; LS, lateral septum; LSD, dorsal lateral septum; LV, lateral ventricle; NS, not significant; PS, photostimulation.

We next sought to dissect the neural circuits underlying ventral hippocampal suppression of food intake with optogenetic approaches. Optogenetics and Cre-lox technology allows for selective manipulation of individual cell types and their projection targets^29^,^30^. To selectively activate glutamatergic projections from vhDG–CA3 neurons, the vHPC of Vglut2-Cre mice were transduced with ChR2 fused to enhanced yellow fluorescent protein (eYFP) (AAV2-EF1α-DIO-hChR2-eYFP) (Fig. 3a,b; Supplementary Fig. 4). Dense ChR2–enhanced YFP (ChR2–eYFP) expressing fibres were observed in the LS of ChR2–eYFP-transduced mice (Fig. 3c; Supplementary Fig. 4). To determine the behavioural relevance of vhDG–CA3 glutamatergic projections to the LS, we inserted a fibre optic cannula above the LS and applied photostimulation (PS; 20 Hz for 1 s; repeated every 4 s for 30 min) to vhDG–CA3 glutamatergic fibres in the LS (Fig. 3a,d). Strikingly, we observed that PS of ChR2-expressing glutamatergic fibres in the LS rapidly reduced food intake (Fig. 3e). We are aware that it is possible that *in vivo* optogenetic stimulation of vhDG–CA3 neuronal fibres in the LS might activate neuronal fibres of passage to additional brain regions or result in antidromic activation of the vHPC to reduce feeding. Further studies are needed to explore these possibilities.

Subsequent ChR2-assisted circuit mapping^31^ was performed in ChR2-transduced mice. As expected, we identified functional glutamatergic synaptic connections from vhDG–CA3 neurons to postsynaptic neurons in the lateral septum (Fig. 3f). Brief presentation of blue light pulses (473 nm; 1–3 ms) to ChR2–eYFP-expressing fibres in the lateral septum consistently evoked synaptic currents in the LS neurons, which were diminished using CNQX (an AMPA receptor antagonist) treatment (Fig. 3f,g). We next examined if the light-evoked synaptic currents at LS neurons were from direct monosynaptic input from vHPC axon terminals or from indirect polysynaptic inputs due to feedback. To test this, we performed similar experiments referred above. We recorded and compared the amplitudes of the light-evoked EPSCs before and after perfusion of the sodium channel blocker tetrodotoxin (TTX; 1μM) and potassium blocker 4-amynopyridine (4-AP; 100 μM) to remove any network activity^32^. We observed that there were no apparent changes in the amplitudes of light-evoked EPSCs (Fig. 3h), suggesting that the light-evoked synaptic currents at LS neurons were likely from direct monosynaptic inputs from vHPC axon terminals. Collectively, these results reveal a neural circuit consisting of ventral hippocampal projections to the lateral septum that are glutamatergic in nature and sufficient to suppress appetite.

**Figure 4:**
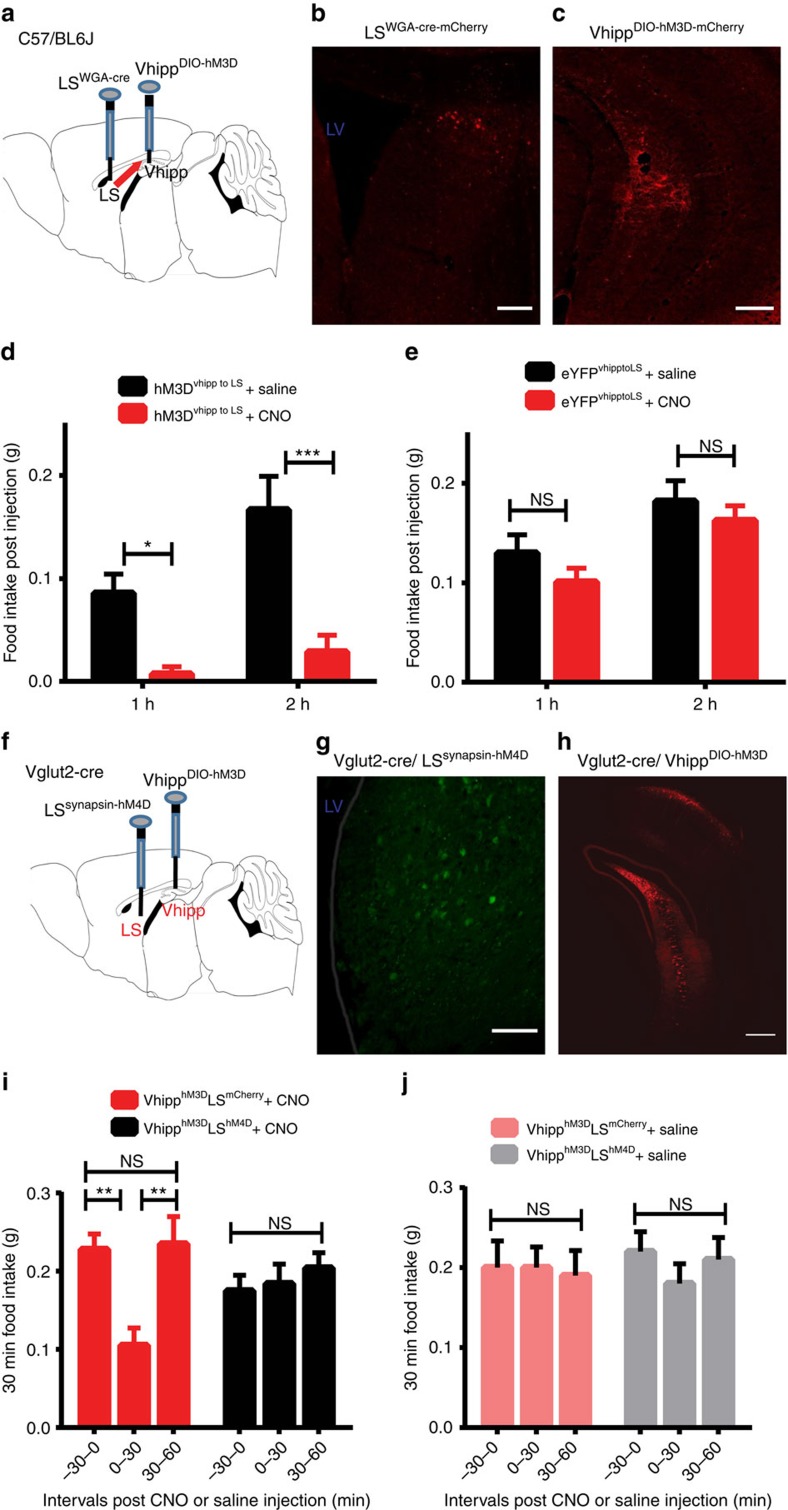
Anatomical characterization of vHPC projections to LS that suppresses appetite. (**a**) Schematic of experimental design. WGA-Cre was bilaterally transduced into lateral septum neurons simultaneously with ventral hippocampal injections of Cre-dependent hM3Dq-mCherry. (**b**) Representative WGA-Cre expression in LS and (**c**) Cre-dependent hM3Dq-mCherry expression in ventral hippocampus. (**d**) Feeding assays demonstrate that selective chemical genetic activation of vHPC→LS-projecting neurons suppressed food intake (*n*=7 mice; F_2,66_=8.058, *P*=0.0007; two-way ANOVA with Sidak's *post hoc* test;). (**e**) Mice injected with WGA-Cre in LS- and Cre-dependent fluorescent protein in ventral hippocampus showed no apparent change in food intake in response to CNO (*n*=5 mice; F_2,84_=0.05225, *P*=0.9491; two-way ANOVA). (**f**) Outline of approach to simultaneously activate vHPC glutamatergic neurons and inhibit lateral septum neurons by expressing hM3Dq and hM4Di in vHPC neurons and LS neurons, respectively. Cre-dependent hM3Dq was bilaterally transduced into the ventral hippocampus of Vglut2-Cre mice together with bilateral injections of hM4Di in the LS. (**g**,**h**) Representative images of hM4Di in LS (**g**) and Cre-dependent hM3Dq in ventral hippocampus (**h**). (**i**) CNO administration (1 mg kg^−1^; i.p.) suppressed food intake in Vhipp^hM3D^: LS^mCitrine^-transduced mice but did not suppress food intake in Vhipp^hM3D^: LS^hM4D^-transduced mice (*n*=5 mice; F_2,54_=3.653, *P*=0.0325; two-way ANOVA with Sidak's *post hoc* test). (**j**) Control saline injections failed to impact food intake in both groups of mice (*n*=5 mice for each group; F_2,36_=0.2845, *P*=0.7540; two-way ANOVA). Data represent mean±s.e.m.; all behavioural experiments and treatment conditions were repeated at least two times per animal; *P<0.05, **P<0.01, ***P<0.001. Scale bars, 150 μm for **d**,**g**; 200 μm for **c;** 300 μm for **h**. ANOVA, analysis of variance; LS, lateral septum; LV, lateral ventricle; NS, not significant.

We next employed a dual AAV vector delivery approach to selectively activate vhDG–CA3 neurons that project to lateral septum. First, we bilaterally transduced LS neurons with an AAV vector expressing the trans-synaptic tracer wheat germ agglutin (WGA) fused to Cre-recombinase (AAV2-EF1α-mCherry-IRES-WGA-Cre) (Fig. 4a,b)^33^,^34^. Ventral hippocampal neurons were simultaneously targeted with a second Cre-dependent AAV vector encoding hM3Dq (Fig. 4a,c), allowing for selective labelling and manipulation of ventral hippocampal neurons that project to the lateral septum^33^,^34^. As expected, CNO administration reduced food intake in the mice transduced with hM3Dq (Fig. 4d and Supplementary Fig. 5e), but not in control mice expressing fluorescent proteins (Fig. 4e and Supplementary Fig. 5d), further demonstrating an anorexigenic neural circuit from vHPC to LS. To expand these findings, we used an identical dual AAV vector delivery approach to express the inhibitory DREADD-hM4Di in vHPC neurons that project to LS. Since we detected an increase in food intake in the light period following inhibition of vHPC glutamatergic neurons (Fig. 1f), we sought to determine if selective inhibition of the LS-projecting vHPC neurons would facilitate food intake in the early-dark period when mice usually consume more. As expected, we observed that selective DREADD-based inhibition of the LS-projecting ventral hippocampal neurons trended towards increasing food intake (Supplementary Fig. 5f).

### Inactivation of LS neurons reduces vHPC reduction of feeding

Next, we evaluated the necessity of LS neurons in mediating ventral hippocampal suppression of food intake. To achieve this, we inactivated LS neurons by targeting the inhibitory DREADD-hM4D_i_ to the LS, while simultaneously activating vhDG–CA3 glutamatergic neurons with the stimulatory DREADD-hM3Dq (Fig. 4f–h). Similar to the results shown in Fig. 1, we found that CNO administration reduced food intake in mice transduced with control mCherry in the LS (AAV2-hSyn-mcherry). However, administration of CNO failed to reduce food intake in mice that were also transduced with hM4D_i_ in the LS (Fig. 4i,j). Together, these results indicate that suppression of appetite following stimulation of vhDG–CA3 glutamatergic neurons is dependent, at least in part, on activation of LS neurons.

### Contribution of BNST to vHPC suppression of food intake

**Figure 5:**
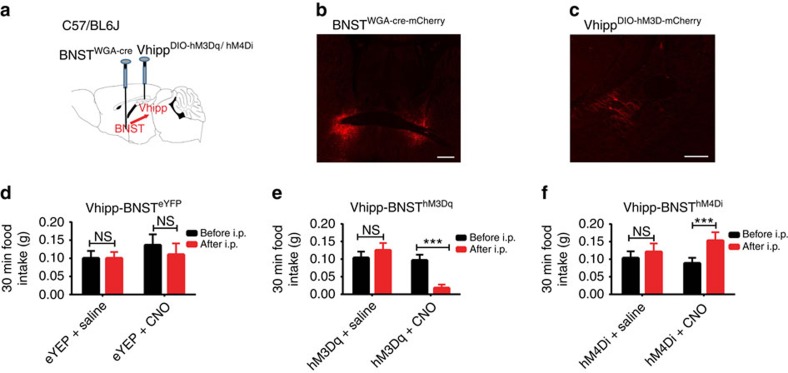
Dissection of the circuit from vHPC to BNST that suppresses appetite. (**a**) Schematic of experimental design. WGA-Cre was bilaterally transduced into BNST neurons. Simultaneously, the ventral hippocampus of the same mice was targeted with a second bilateral injection of Cre-dependent hM3Dq, hM4Di or control eYFP vectors, respectively. (**b**,**c**) Representative images obtained from a transduced mouse showing WGA-Cre-mCherry expression in BNST neurons (**b**) and hM3Dq expression in the ventral hippocampus (**c**). (**d**–**f**) Feeding behaviour assays indicating that administration of CNO (1 mg kg^−1^) did not affect feeding behaviour in mice transduced with WGA-Cre in BNST and control eYFP in vHPC (**d;** *n*=4; paired Student's *t*-test, *t*=2.03, df=7, *P*=0.08). Selective hM3Dq-induced activation of vHPC→BNST-projecting neurons reduced food intake (**e;** *n*=9 mice; paired Student's *t*-test, *t*=4.05, df=13, *P*=0.001); while hM4Di-mediated inhibition of BNST-projecting vHPC neurons increased food intake (**f**; *n*=6 mice; paired Student's *t*-test, *t*=5.01, df*=*10, *P*=0.0005)). For all feeding assays shown, 30 mins of baseline food intake was measured prior to i.p. injections (before i.p.) of saline or CNO (1 mg kg^−1^). Food intake was subsequently measured 30 mins following i.p. injections (after i.p.). Data represent mean±s.e.m.; all behavioural experiments and treatment conditions were repeated at least two times per animal; ***P<0.001. Scale bars, 500 μm for **b** and 200 μm for **c**. ANOVA, analysis of variance; NS, not significant.

In addition to studying the roles of lateral septum to ventral hippocampal suppression of food intake, we also studied the capability of the bed nucleus of stria terminalis (BNST) to mediate ventral hippocampal suppression of feeding since emerging evidence demonstrates that BNST participates in orchestrating feeding behaviours. Previous studies have revealed that BNST regulates food intake by receiving synaptic inputs originating from the arcuate nucleus and by projecting synaptic inputs to the lateral hypothalamus^35^,^36^. To test the potential involvement of BNST in vHPC regulation of food intake, as described in the experiments performed on the LS (Fig. 4a–e), bilateral expression of WGA-Cre in BNST neurons and subsequent Cre-dependent expression of hM3Dq in vHPC neurons were achieved in wild-type mice (Fig. 5a–c). We observed that expression of hM3Dq was confined to a small region of the vHPC (Fig. 5c). I.p. injections of CNO did not exert apparent effects on feeding in control eYFP-transduced mice but significantly reduced food intake in hM3Dq-transduced mice (Fig. 5d,e; Supplementary Fig. 5g and h). We next performed analogous loss-of-function experiments by transducing the inhibitory DREADD-hM4Di into ventral hippocampal neurons in the WGA-Cre-transduced mice in the BNST. As expected, food intake was increased in response to CNO injections (Fig. 5f). Taken together, these results demonstrate that BNST also contributes to ventral hippocampal regulation of food intake.

## Discussion

In the present study, we dissected two putative neural circuits underlying ventral hippocampal suppression of food intake by using approaches that included chemogenetics, optogenetics, retrograde tracing and electrophysiology. These approaches allowed us to overcome long-standing technical limitations to study the functional roles of vHPC in feeding within the specificity of individual neural circuits.

The vHPC is classically associated with emotional behaviour and memory^6^,^7^,^8^,^9^. Strikingly, several lines of evidence indicate that the vHPC influences feeding behaviour by regulating learned or motivational aspects of food intake^11^,^12^,^13^,^14^,^15^,^19^,^20^,^21^. Previous research has utilized pharmacological or chemical lesion studies to determine that the hippocampus and, in particular, its ventral pole suppresses appetite^13^,^14^,^15^. In addition, elegant studies have outlined the functions of classical satiety and orexigenic hormones in hippocampal synaptic plasticity and feeding behaviour^16^,^17^,^18^,^19^,^20^,^21^. However, the cell types and specific neural circuits underlying the hippocampus role in feeding have remained elusive as previous techniques were not cell-type specific. In this study, we find that chemogenetic activation of excitatory glutamatergic neurons localized in the vHPC is sufficient to decrease food intake (Fig. 1). These neurons were mainly restricted to area CA3 and DG of the vHPC, although we cannot rule out sparse activation of neurons in CA1 (Supplementary Fig. 6). Further work is needed to determine the specificity of different vHPC subregions, such as CA3, DG and CA1, in the regulation of feeding since these different subregions exert different roles in other behaviours, such as anxiety and learning^6^,^37^. As an important control, DREADD-induced activation of vHPC neurons did not exert apparent effects on animals' physical activity and induced only modest increases in anxiety, indicating that the observed changes in appetite were not secondary to the alterations in locomotion or anxiety but from the underlying feeding circuits (Fig. 2; Supplementary Fig. 3).

Meanwhile, we are aware that a previous study^24^ reported that optogenetic activation of vHPC DG neurons decreased anxiety, contrasting to the results in this project and the study from another group^38^ showing that optogenetic activation of excitatory basolateral amygdala inputs to the vHPC increased anxiety. These seemingly inconsistent findings could be reconciled by the fact that the optogenetic stimulation paradigm in the study^24^ was previously shown to result in feed-forward inhibition of hippocampal area CA3 (refs 39, 40), which we stimulated in our present study (Supplementary Fig. 6). Together, the anxiety states largely depend on the stimulation protocols and brain regions.

Further studies indicated that activation of vHPC glutamatergic projections to the lateral septum is sufficient to exert anorexic effects on feeding. For example, optogenetic and chemical genetic activation of LS-projecting ventral hippocampal neurons suppressed food intake (Figs 3 and 4), and inactivation of the neurons within lateral septum occluded hippocampal suppression of food intake (Fig. 4). Together, these results suggest that the vHPC exerts anorexigenic effects on feeding by modulating the neuron activities in the lateral septum. Interestingly, it is well-demonstrated that the lateral septum relays hippocampal information to brain regions involved in the regulations of feeding behaviour, such as paraventricular hypothalamus, medial hypothalamus and ventral tegmental area^35^,^41^,^42^,^43^. These septal inputs may facilitate the communication of emotional information, represented in the vHPC, to key brain regions involved in motivational behaviours. Thus, stimulation of vHPC→LS glutamatergic projections may provide input to hypothalamic or midbrain neurons via the LS. Such input is well-suited to modulate feeding behaviour by providing the relevant emotional and cognitive information encoded within the vHPC. Future studies will dissect the neural circuits originating from the lateral septum that project to hypothalamic and midbrain regions.

We also find that BNST neurons contribute to ventral hippocampal suppression of food intake (Fig. 5; Supplementary Fig. 5g,h). Meanwhile, BNST neurons project to hypothalamic brain regions^36^,^44^,^45^ and the ventral tegmental area^46^,^47^, brain regions responsible for anxiety and motivational behaviours^44^,^45^,^46^,^47^. Consistently, it is demonstrated that subsets of BNST neurons and their neuronal projections contribute to the control of motivation states^47^ and anxiety behaviour^46^. Strikingly, emerging evidence also implicates potential interactions between feeding, anxiety and stress^48^,^49^. Further studies are needed to identify the precise neural circuits governing this complicated and often paradoxical relationship.

In this study, we focused on investigating the functional roles of neurons localized in the DG and CA3 regions of the vHPC. It is likely, however, that additional ventral hippocampal circuits are capable of exerting modulatory influences over feeding behaviour. For example, it was recently shown that ventral subiculum/CA1 projections to nucleus accumbens, amygdala and prefrontal cortex can modulate a variety of behavioural functions including: anxiety, approach/avoidance, addiction, spatial working memory and susceptibility to depression^25^,^26^,^50^,^51^,^52^. The function of ventral CA1 projections in feeding behaviour is another active area of research that remains to be determined.

Meanwhile, the chemogenetic and optogenetic approaches used in this study offer exceedingly useful strategies to assign behavioural functions to specific neurons and neural circuits. However, exogenous activation and inactivation do not provide information about the activity level and likely function of selective neurons or neural circuits during naturally occurring behaviours. It will be important for future studies to determine how vHPC neuron activity is altered during feeding or appetitive behaviours. Advances in two-photon calcium imaging and genetically encoded calcium indicators make it feasible to selectively determine how activity in these neurons is altered during changes in physiology and metabolic status, a top priority for future studies^53^,^54^,^55^. Collectively, effectively seeking food in a complex environment requires the ability to associate and remember contexts that involve food. Furthermore, neural mechanisms allowing an animal to suppress food intake during conditions when feeding is unfavourable would be evolutionarily advantageous. The functional anatomy of the hippocampal formation is ideal for coordinating the flexible mental representations needed to regulate feeding behaviour under such changing environmental conditions^5^,^11^,^12^,^13^. We propose that ventral hippocampal neurons possess the ability to coordinate an adaptive feeding behaviour response by providing emotional and cognitive input to key motivational centres, such as the LS. Such a neural structure would be ideal for maintaining energy homeostasis during changing metabolic needs and environmental conditions, and may also serve as a potential therapeutic target in the treatment of appetite disorders, such as anorexia nervosa.

## Methods

All experiments were performed in agreement with the guidelines described by the US National Institute of Health's Guide for the Care and Use of Laboratory Animals and were approved by the Institutional Animal Care and Use Committee at State University of New York Upstate Medical University. Food deprivation was from 1800 to 0900 hours.

### Mice

Adult animals (5- to 8-week old) were housed on a 12-h light/dark cycle and provided *ad libitum* access to water and standard rodent chow (LabDiet; 5008 Formulab Diet). In most cases, behavioural experiments were performed on male mice. The strains of mice used in experiments included: Vglut2-IRES-Cre and C57/BL6J (Jackson Laboratory). Prior to stereotaxic surgical injections, mice were housed in groups. Following surgical procedures, all animals recovered for at least 2 weeks and were subsequently single caged for behavioural experiments. To habituate to behavioural assays, mice were handled twice daily for 1 week prior to all behavioural experiments. Animals were randomly assigned to control (animals transduced exclusively with fluorescent proteins) or experimental groups (animals transduced with DREADD-hM3Dq, DREADD-hM4Di or ChR2) and statistical analysis was conducted following the conclusion of behavioural experiments.

### Viral vectors

The viral vectors used in this study included: AAV vectors for Cre-dependent expression of ChR2 (AAV2-EF1α-DIO-hChR2(H134R)-EYFP) or eYFP (AAV2-EF1α-DIO-EYFP) and trans-synaptic tracer virus (AAV2-EF1α-mCherry-IRES-WGA-Cre) were provided courtesy of K. Deisseroth. AAV vectors expressing Cre-dependent hM3Dq, hM4Di or mCherry (AAV2-hSyn-DIO-hM3D(Gq)-mCherry; AAV2-hSyn-DIO-hM4D(Gi)-mCherry; AAV2-hSyn-DIO-mCherry) and AAV vectors expressing non-Cre-dependent DREADDs or control fluorescent proteins (AAV2-hSyn-HA-hM4D(Gi)-IRES-mCitrine, AAV2-hSyn-mcherry) were provided courtesy of B. Roth. Viral vectors were provided by the UNC viral core facility, and on arrival were aliquoted and stored at −80 °C prior to stereotaxic injections.

### Viral injections and fibre placement

Prior to surgical procedures, mice (5 to 8-week old) were anaesthetized with i.p. injections of a mixture of ketamine/zylazine (60–75 mg kg^−1^/10 mg kg^−1^; VET One) and placed in a stereotaxic frame (Stoelting). One or two small burr holes were made directly above the viral injection sites unilaterally or bilaterally, as described in the text, using a micro-precision drill (CellPoint Scientific). A micromanipulator (Narishige) delivered controlled viral injections into the vHPC (bregma−3.4 mm; midline±2.2 mm; dorsal surface 2.5 mm), lateral septum (bregma+0.4 mm; midline+0.4 mm; dorsal surface −2.0 mm) and BNST (bregma+0.2 mm; midline+0.9 mm; dorsal surface−3.5 mm) at a rate of 0.2 μl min^−1^. Injection needles were left in place for 10 mins following all injections to assure adequate viral delivery. Injection volumes varied for each targeted region as follows: vHPC (0.5 μl), lateral septum (0.3 μl) and BNST (0.3 μl). For trans-synaptic viral injections, two stereotaxic injections were performed. Trans-synaptic tracer virus (AAV2-EF1α-mCherry-IRES-WGA-Cre) was targeted to the projection region of interest (LS or BNST) simultaneously with a second AAV injection in the vHPC (Cre-dependent hM3Dq or eYFP). For the experiments performed with *in vivo* photostimulation, following viral injections, ferrule-capped fibres (1.25 mm Ceramic Ferrule; Thorlabs) were implanted 0.5 mm above the lateral septum (bregma+0.4 mm; midline+0.4 mm; dorsal surface—1.5 mm). Grip cement (DENTSPLY) was used to fix the ferrule-capped fibres to the skull. After surgery, mice were allowed 2 weeks for expression of viral proteins and recovery from surgery before beginning behavioural experiments.

### Pharmacology

Picrotoxin (GABA_A_ receptor antagonist; 50 μM), CNQX (AMPA receptor antagonist; 10 μM); TTX (1 μM) and 4-AP (100 μM) were purchased from Sigma. CNO was purchased from Enzo Life Sciences. Pharmacological agents were applied via i.p. injections for behavioural experiments or via bath addition for electrophysiological experiments.

### Electrophysiology and circuit mapping

Acute coronal sections of the lateral septum were prepared from mice transduced with ChR2–eYFP in the vHPC. Mice were deeply anaesthetized with isoflurane and decapitated. Mouse brains were dissected rapidly and placed in ice-cold oxygenated (95% O_2_ and 5% CO_2_) solution containing (in mM): 110 choline chloride, 2.5 KCl, 1.25 NaH2PO4, 2 CaCl2, 7 MgSO4, 25 D-glucose, 3.1 Na-pyruvate and 11.6 Na-L-ascorbate, pH 7.3. Coronal brain slices (200-μm thickness) were cut with a vibratome (Vibratome 1000S) and maintained in an incubation chamber at 34 °C for 30 min, and then brought to room temperature until transferred to a recording chamber. During experiments, an individual slice was transferred to a submersion-recording chamber and continuously perfused with recording solution containing the following (in mM): 119 NaCl, 25 NaHCO_3_, 11 D-glucose, 2.5 KCl, 1.25 MgCl_2_, 2 CaCl_2_ and 1.25 NaH_2_PO_4_, aerated with 95% O_2_/5% CO_2_ (1∼2 ml min^−1^ at room temperature). Recordings were made at levels throughout the lateral septum. Neurons were identified using a Nikon microscope and ChR2–eYFP-expressing fibres were observed by fluorescence emission. Whole-cell patch-clamp recordings were made on neurons in lateral septum using electrodes with tip resistances 3–5 MΩ. Recording pipettes were routinely filled with a solution containing (in mM): 125 K-gluconate, 15 KCl, 10 Herpes, 8 NaCl, 4 Mg-ATP, 0.3 Na-GTP, 10 Na_2_-phosphocreatine and 2 EGTA (pH 7.30). The holding potential for voltage-clamp recordings was −70 mV, and responses were digitized at 10 kHz through whole experiments using Axo-patch 700B amplifier analysed with pClamp 10.0 Software (Molecular Devices, CA). Neurons with series resistances <30 MΩ were used for recordings. The postsynaptic currents (PSCs) were recorded, while shining blue light on the surface of brain slices using an optic fibre connected to a blue laser power (CrystaLaser 473 nm). The PSCs were elicited by delivering light pulses ranging from 0.1 to 1 mW at the specimen every 20 s. Light pulse (duration at 1 to 3 ms) was controlled by pCLAMP 10 software. In most cases, recordings were initiated 5 min after the whole-cell configuration.

### Feeding behaviour assays

*In vivo chemical genetic manipulation*. After 2 weeks for recovery from surgical procedures, mice were individually housed in home cages. All animals were provided *ad libitum* access to standard chow food and water, unless otherwise noted. All behavioural experiments were conducted in home cages as described except where noted. The week prior to starting experiments, mice were handled twice daily to habituate to behavioural paradigms. During feeding behaviour experiments, food was replaced daily with ∼20 g of fresh standard chow. Food intake was manually calculated in the home cage at the indicated time points by briefly removing the food from the hopper and obtaining its weight. Food intake was measured at multiple ascending time points, as described in the text, in response to i.p. injections of CNO (1 mg kg^−1^; prepared in sterile 0.9% saline solution; 200 μl) or saline (0.9%; 200 μl). For acute feeding assays, the administrations of vehicle saline and CNO were following the order of Day 1 and Day 2 with saline, Day 3 with CNO, Day 4 with saline and Day 5 with CNO. Average food intake during saline and CNO conditions were then calculated by averaging the food intake for both conditions across all trials, as we performed in our previous study^27^. Acute food intake measurements were performed during both the light (1030–1530 hours) and dark period (2030–2200 hours). For chronic feeding behaviour experiments, twice daily i.p. injections of saline or CNO (1 mg kg^−1^) were given at 0900 and 1800 hours, and food weight was measured daily at 1800 hours. To perform overnight fasting experiments, all food was removed from home cages at 1800 hours. At 0900 hours the following morning, immediately prior to i.p. injections, ∼20 g of fresh standard chow was returned to the food hopper of the mouse's home cage. Food intake was manually calculated at the indicated time points, as described in the text, in response to i.p. injections of CNO (1 mg kg^−1^). Three days were allotted between experimental trials to ensure that animals recovered from food deprivation. All behavioural experiments were repeated at least twice per mouse for each experimental condition.

*In vivo optogenetic manipulation*. After 2 weeks of recovery from surgical procedures, fibre optic cables (200 μm diameter core; BFH48-200-Multimode, NA 0.48; Thorlabs) were individually attached to implanted fibre optic cannulas (1.25 mm Ceramic Ferrule) via ceramic mating sleeves (Thorlabs). Following fibre optic attachment, mice were individually housed in modified home cages which allowed free range of movement. Prior to behavioural experiments, all mice were habituated to home cages for 1 week. *Ad libitum* access to standard rodent chow and water was made available prior to initiating experimental sessions. During PS, 30 mins of light pulse trains (20 pulses for 1 s; pulse duration at 3 ms, repeated every 3 s for 30 mins) were applied using a waveform generator (Agilent; 3,322 A 20MHz Function Waveform Generator) that provided output to a blue laser power (473 nm; Altechna). Light power exiting the fibre tips was calculated prior to behavioural experiments to be 15–20 mW. At the onset of *in vivo* PS experiments, three to four food pellets (10–12 g of food) were placed on the floor of the home cage. Food intake was calculated by weighing the food amount following 30 mins with or without PS, as described in the text. All behavioural experiments were repeated at least twice per mouse. Following behavioural experiments, mice were perfused and the location of viral infection and fibre optic placement was confirmed with light microscopy.

### Open field behavioural testing

Virally transduced mice were habituated to the behavioural room for 30 mins prior to beginning experimental sessions. The open field consisted of a brightly lit, 500 mm^2^ arena. The centre of the arena was scored as the 250 mm^2^ area in the centre of the open field. Ten minutes before placing the mice in the arena, i.p. injections of CNO (1 mg kg^−1^) or control vehicle saline were administered. Exploratory behaviour was recorded and analysed for 10 or 30 mins using ANY-maze software (Stoelting). Between trials, the arena was cleaned with 70% ethanol. The first trial consisted of i.p. injections of CNO on Day 1, followed by i.p. injections of vehicle saline on Day 2. Two weeks later, a second trial was conducted where vehicle saline was administered on Day 1 and CNO was administered on Day 2. The average total distance travelled, time spent in the centre and per cent distance travelled in the centre of the arena was calculated for each mouse by averaging the data from both experimental trials. Per cent distance travelled in the centre of the arena was determined by dividing the distance travelled in the centre by the total distance that the mouse travelled during 10 or 30 mins of exploration.

### Immunofluorescence and imaging

Following behavioural experiments, mice were anaesthetized with ketamine/zylazine (60–75 mg kg^−1^; 10 mg kg^−1^) and perfused with 0.1 M phosphate-buffered saline (PBS), followed by 4% paraformaldehyde in 0.1 M PBS. Brains were extracted and post-fixed for 24–48 h at 4 °C in the same fixative used for perfusion. Brain samples were sequentially incubated in 10, 20 and 30% sucrose solutions (prepared in 0.1 M PBS) for 24 h at 4 °C. Preserved brain samples were sectioned (40 μm), using a cryostat (Leica Microsystems) and mounted on glass slides for immunofluorescence imaging.

DREADD-based *in vivo* neuronal activation was confirmed by *post hoc* Fos quantification. After behavioural experiments, mice were i.p. injected with CNO or saline 30 mins prior to perfusion. Expression of DREADD-hM3Dq was confirmed by the expression of hM3Dq-mCherry. To determine Fos signals, brain sections were washed three times in 0.1 M PBS for 5 mins at room temperature. Sections were then blocked for 2 h in 2% bovine serum albumin (Sigma-Aldrich; Lot 084K8926) in 0.1 M PBS and 0.1% Tween-20. Primary antibody (rabbit anti-Fos; 1:150; Santa Cruz Biotechnology) incubated in blocking solution was added to sections at 4 °C for 18–24 h. Sections were then washed three times for 5 mins each in 0.1 M PBS and incubated in secondary antibody (donkey anti-rabbit 680, 1:2,000; Life Technologies) for 2 h at room temperature. Sections were again washed three times with 0.1 M PBS and mounted onto glass slides (Fisherbrand Superfrost Plus Microscope Slides). Slides were stored at 4 °C prior to image acquisition.

Quantification of Fos and co-localization of Fos and mCherry were conducted on 40-μm vHPC-containing brain slices. Florescent images were collected on either a Nikon epiflourescent microscope, Zeiss LSM 510 Meta confocal microscope or Zeiss LSM 780 confocal microscope. Images were minimally processed for optimal characterization and quantification of data. All images were processed identically to avoid artificial representations of data. Confocal images of 15–20 slices per mouse for ventral hippocampal sections were collected using × 20 and × 40 objectives, and neurons expressing hM3Dq-mCherry and/or Fos were counted in a single section. Total number of mCherry or Fos-positive cells was manually counted from a random subset of experimental (CNO) and control (saline) slides. mCherry-positive cells in the vHPC were considered Fos positive if the Fos signal clearly overlapped with neuronal soma expressing mCherry.

### Statistical analysis

Animals were excluded from samples if *post hoc* histological analysis showed inaccurate AAV viral injections or cannula placements. All data were analysed using Prism 6.0 (GraphPad Software) with appropriate statistical tests for different panels in the figures and figure legends.

## Additional information

**How to cite this article:** Sweeney, P. & Yang, Y. An excitatory ventral hippocampus to lateral septum circuit that suppresses feeding. *Nat. Commun.* 6:10188 doi: 10.1038/ncomms10188 (2015).

## Supplementary Material

Supplementary Information Supplementary Figures 1-6
