# Supplementary material for: An excitatory ventral hippocampus to lateral septum circuit that suppresses feeding
Source: Nat Commun. 2015 Dec 15;6:10188. doi: 10.1038/ncomms10188 (PMC4682174; doi:10.1038/ncomms10188)
Supplement: Supplementary Information — Supplementary Figures 1-6 [file ncomms10188-s1.pdf]

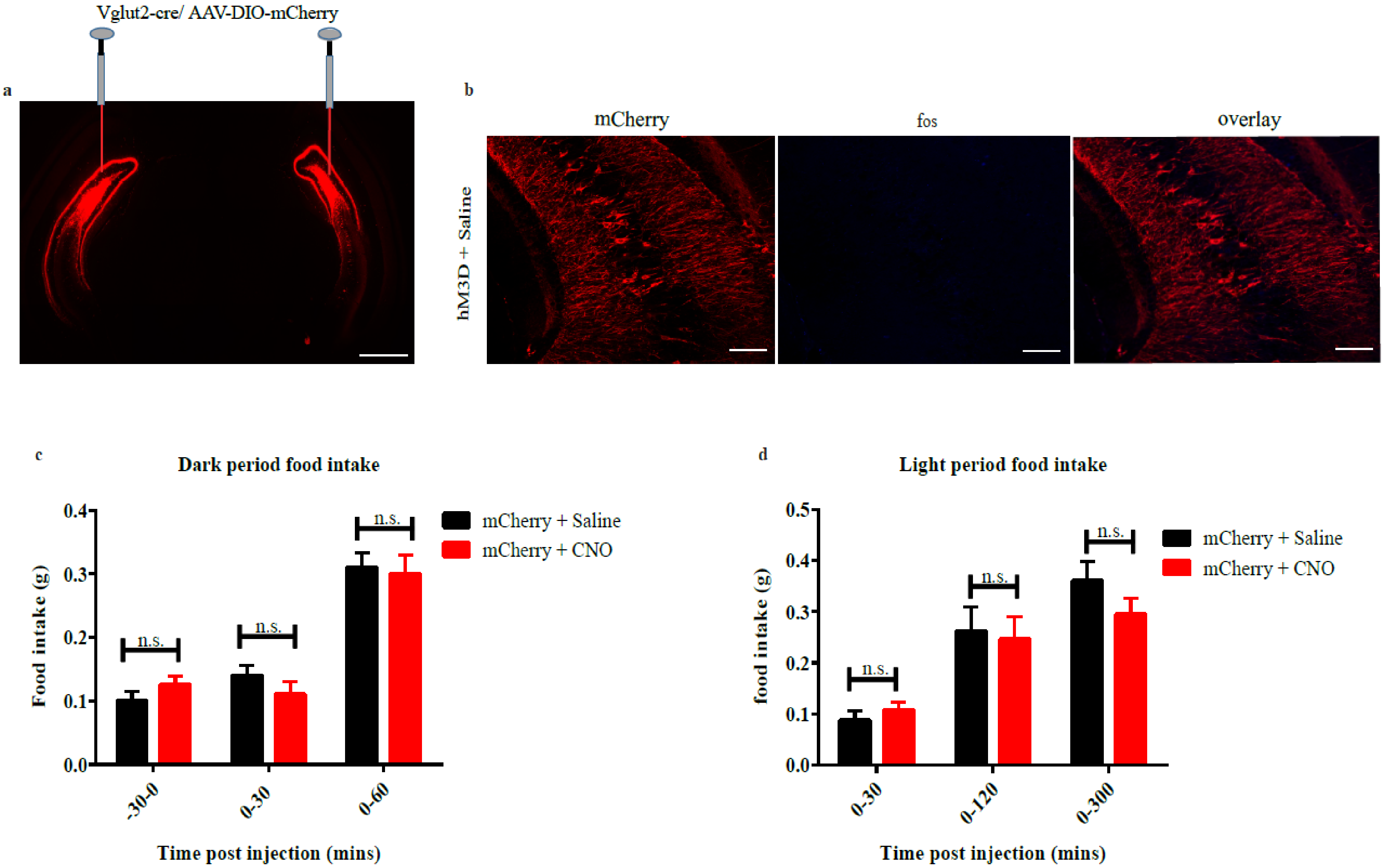

Supplementary figure 1

Supplemental Figure 1| CNO did not reduce food intake in control mCherry-transduced VGlut2-Cre mice. (a) Representative images of bilateral expressions of control mCherry in the ventral hippocampus of a VGlut2-Cre mouse. (**b**) Sample images of CA3 region hM3Dq-mCherry expression (left panel), Fos expression (middle panel), and overlay (right panel). Intraperitoneal injections of saline were given 30 minutes prior to perfusion and brains were subsequently prepared for Fos immunohistochemistry (see methods section). Vehicle saline injections did not increase Fos signals when compared to CNO injections (**Fig. 1c and d). (c and d)** CNO or saline injections did not exert apparent changes in food intake in control mCherry-transduced mice (Two-way ANOVA; Dark period **c:** n = 5 mice for each group; $F_{2, 84} = 1.181$, $p = 0.3119$; left panel; Light period **d:** n = 5 mice for each group; $F_{1, 88} = 0.2829$, $p = 0.5962$; right panel). Data represents mean ± SEM. All behavioral experiments and treatment conditions were repeated at least two times per animal. n.s (not significant), FI (food intake). Scale bars, 1 mm and 50 μm for **a** and **b**, respectively.

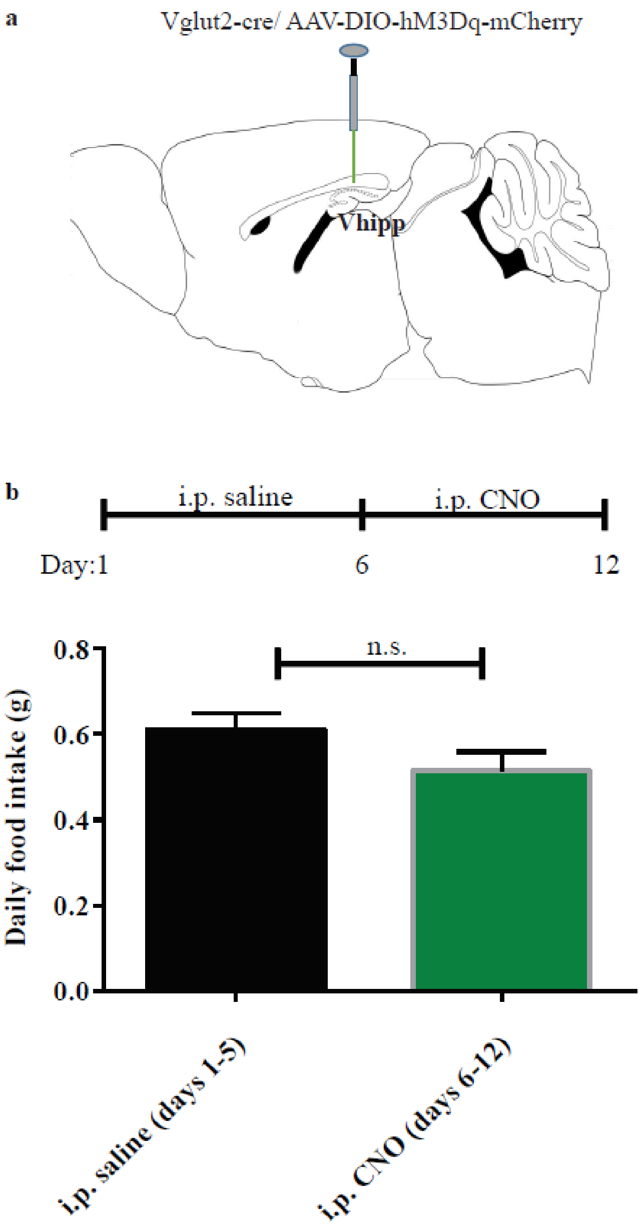

Supplementary figure 2

Supplemental Figure 2| DREADD-based activation of vHPC glutamatergic neurons does not reduce daily food intake. (**a**) Diagram outlining method to transduce vHPC glutamate neurons with DREADD-hM3Dq in Vglut2-Cre mice. (**b)** Top panel, experimental timeline, the hM3Dq transduced mice received intraperitoneal injections of saline (twice per day; 200 μ l) from experimental days 1 to 5, and CNO (twice per day; 1 mg/kg) from days 6 to 12. Bottom panel, grouped behavioral data showing average daily food intake (n = 6; paired Student's t-test, t = 1.72, *df* = 19, p = 0.10). Data represents mean ± SEM for each experimental time-point (as outlined in top panel). n.s. (not significant).

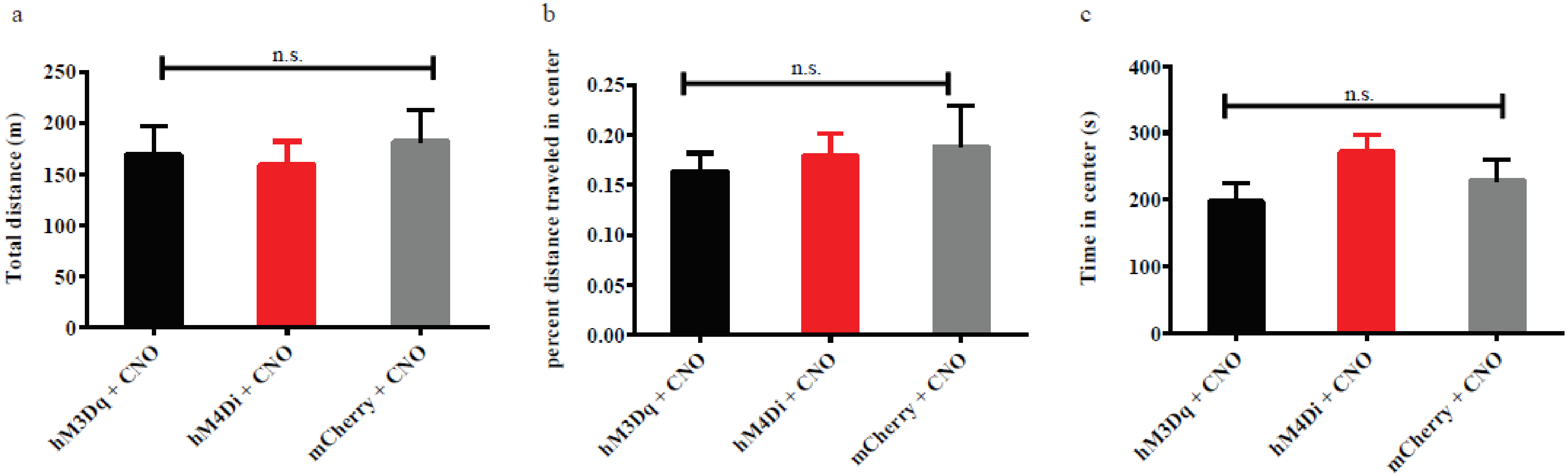

Supplementary figure 3

Supplemental Figure 3| Activation or inhibition of vHPC neurons does not affect locomotion or anxiety after thirty minutes of open field exploration. The stimulatory DREADD-hM3Dq (n=5), the inhibitory DREADD-hM4Di (n=5), or control mCherry (n=5)-transduced Vglut2-Cre mice in vHPC glutamatergic neurons, as previously described **(Fig. 1),** were administered i.p. injections of CNO (1mg/kg) and monitored for thirty minutes during open field exploration to assay for DREADD-induced changes in anxiety or locomoter activity. **(a)** Total distance traveled during thirty minutes of open field exploration. No significant differences were found between CNO treatment conditions (One-way ANOVA; $F_{2, 9}$= 0.16, P=0.85). **(b and c)** No significant differences were found between CNO treatment conditions in percent distance traveled in the center (**b,** One-way ANOVA; $F_{2, 9}$= 0.18, P=0.84) or time spent in the center (**c;** One-way ANOVA; $F_{2, 8}$= 1.82, P=0.22). Intra-peritoneal injections of CNO (1 mg /kg) and saline were administered on consecutive days ten minutes prior to placing animals in the open field. Open field experiments were repeated two weeks later in the opposite order and average values were calculated for each mouse. n.s. (not significant).

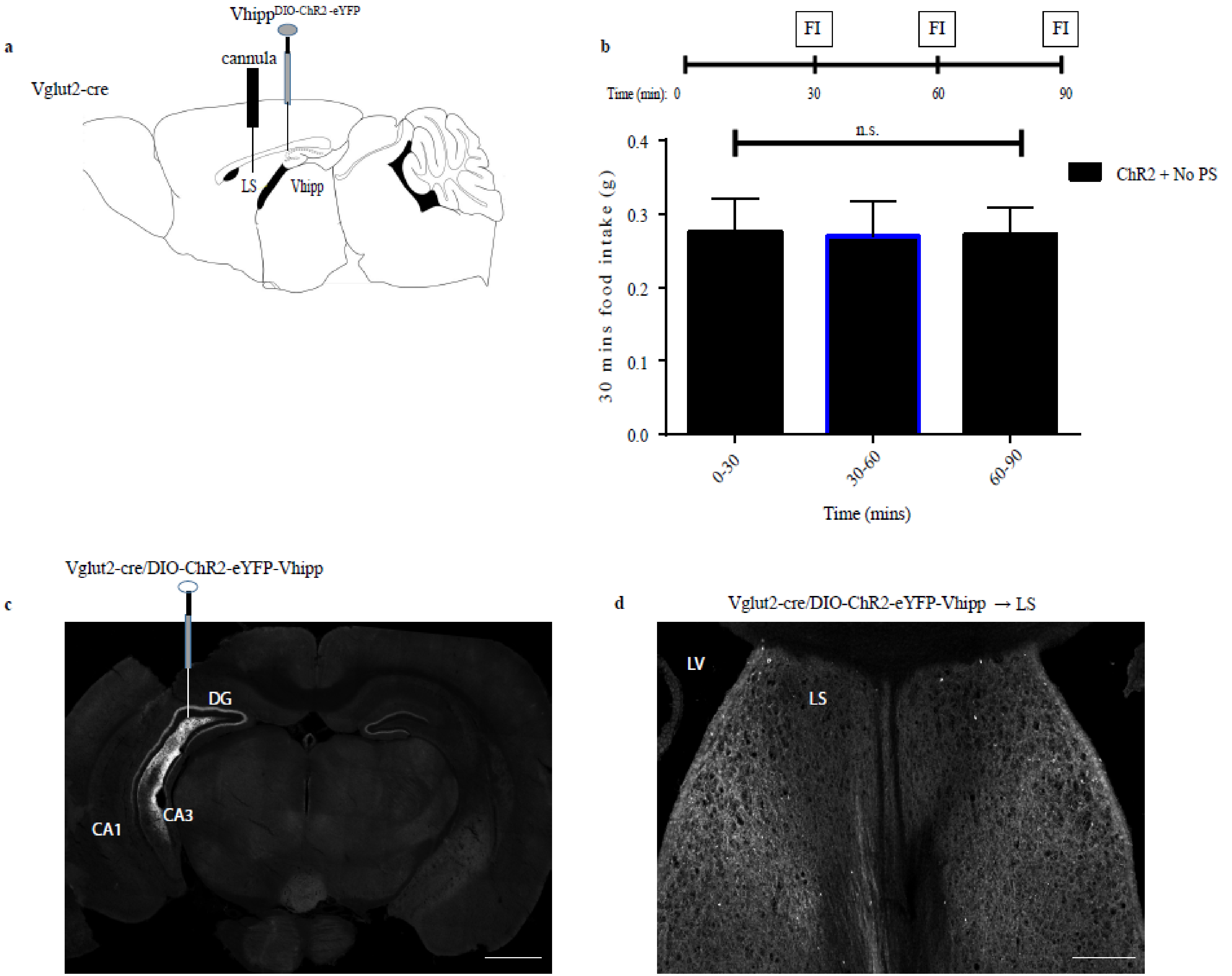

Supplementary figure 4

Supplemental Figure 4| Expression of ChR2 did not exert non-specific effects on food intake without photostimulation. (**a**) Experimental strategy used to express ChR2 in ventral hippocampus and insert fiber optic cannula above the lateral septum. (**b)** Timeline of behavior experiments (top panel) and behavioral data (bottom panel) indicating that ChR2-transduced and cannulated mice showed no observable differences in food intake at all measured time-points (n=8 mice per group). Food intake was measured every thirty minutes without photo-stimulation, as shown. **(c)** Representative sample image showing unilateral transfection of Cre-dependent ChR2 in a Vglut2-Cre mouse. Viral infection was primarily localized to areas DG and CA3 of the ventral hippocampus, as shown. **(d)** Sample image showing ChR2 expressing ventral hippocampal fibers localized in the lateral septum. ChR2-eYFP expressing fibers were detected on both sides of the lateral septum. However, fiber density was noticeably stronger on the ChR2-eYFP injected side than the contralateral side, suggesting that ventral hippocampus bilaterally projects to LS in the two hemispheres. Data represents mean ± SEM. Experiment repeated two times per mouse. LS (lateral septum), LV (lateral ventricle), FI (food intake), n.s. (not significant), DG (dentate gyrus), CA1 (cornus ammonis area 1), CA3 (cornus ammonis area 3). Scale bars, 1mm for **c** and 250µm for **d**.

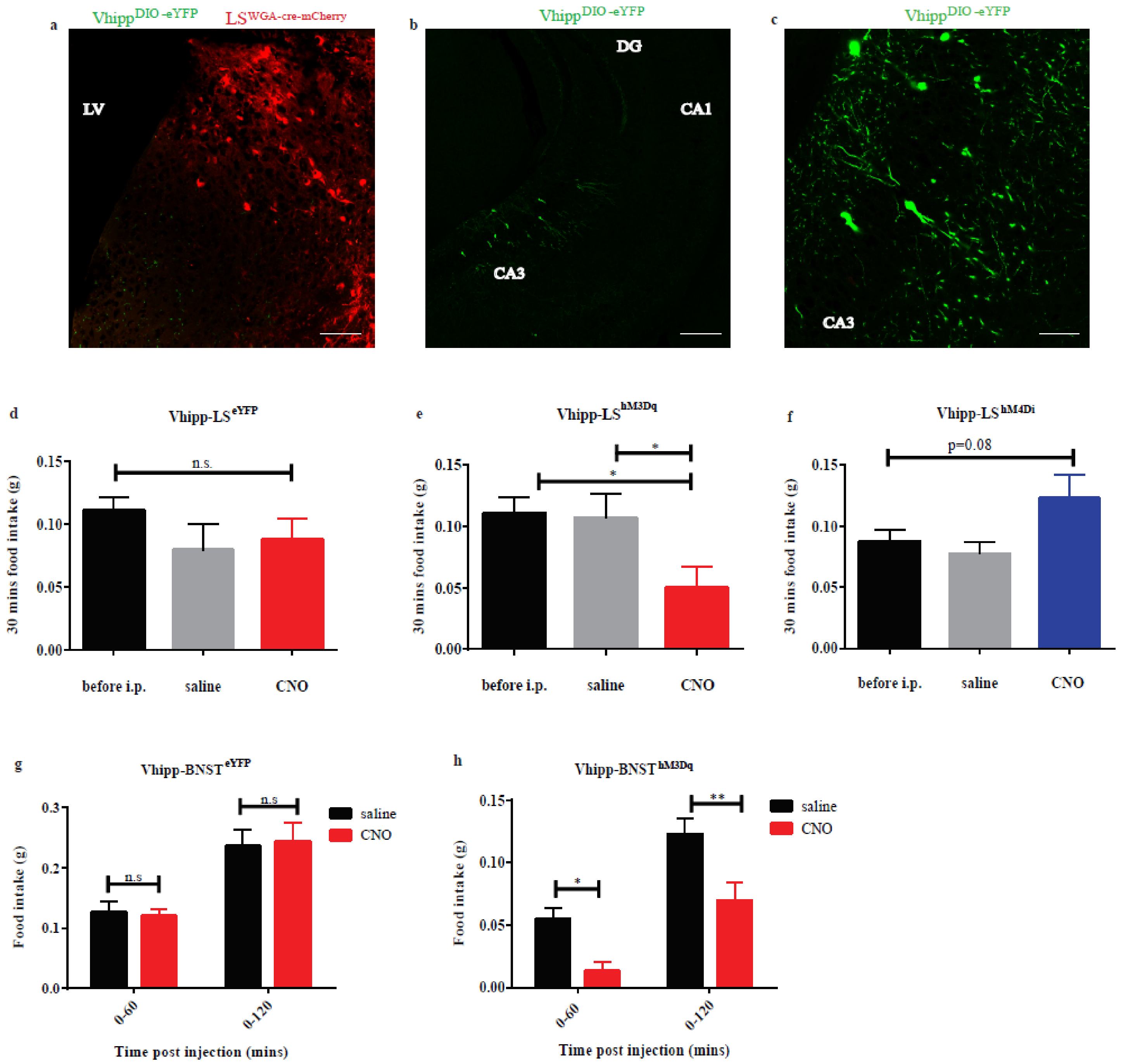

Supplementary Figure 5

Supplemental Figure 5| Trans-synaptic dissection of vHPC projections to LS and BNST WGA-Cre-mCherry (red) was bilaterally injected into LS or BNST together with simultaneous bilateral injections of hM3Dq, hM4Di, or eYFP (green) into the vHPC. **(a and b)** Representative sample images showing WGA-Cre-mCherry transfection in LS (**a)** and eYFP expression in the ventral hippocampus (**b)**. **(c)** Zoomed in panel showing eYFP positive soma and fiber expression in the ventral hippocampus. **(d)** Feeding behavior assays indicating that administration of CNO (1mg/kg) did not affect feeding in mice targeted with WGA-Cre-mCherry in LS and control Cre-dependent-eYFP in vHPC (n=4; paired Student's t-test $F_{2, 44}$= 1.232, p=0.30). (**e**) Selective hM3Dq-induced activation of vHPC→ LS projecting neurons reduced food intake thirty minutes following i.p. CNO injections (n = 7 mice; $F_{2, 53}$= 3.94, P=0.03). (**f**) DREADD-hM4Di induced inhibition of LS projecting vHPC neurons trended towards increasing food intake (n= 6 mice; paired Student's t-test; $F_{2, 65}$= 2.59, p= 0.08). Thirty minutes of baseline food intake was measured prior to all i.p. injections. Food intake was subsequently measured thirty minutes following i.p. injections of saline or CNO (1mg/kg). (**g)** Feeding behavior experiments showing that CNO administration did not impact food intake in mice transduced with WGA-Cre in BNST and Cre-dependent fluorescent protein in the ventral hippocampus (n = 5 mice per group; Two-way ANOVA; $F_{2, 84}$ = 0.07476, p = 0.9280). **(h)** DREADD-hM3Dq induced activation of vHPC→ BNST neurons reduces food intake 1 hour and 2 hours following CNO administration (1 mg/ kg; Two-way ANOVA with Sidak's post hoc test; n = 9 mice per group; $F_{1, 129}$ =18.34, $p <$ 0.0001). Intra-peritoneal injections of saline or CNO (1mg/kg) were administered at the start of behavior experiments. Food intake was manually accessed at 1 and 2 hours following i.p. injections, as shown. Data represents mean ± SEM. All experimental conditions were repeated at least two times per mouse. * $p < 0.05$, **$p < 0.01$.) Scale bars, 600um for **b,** 100um for **a** and **c.**

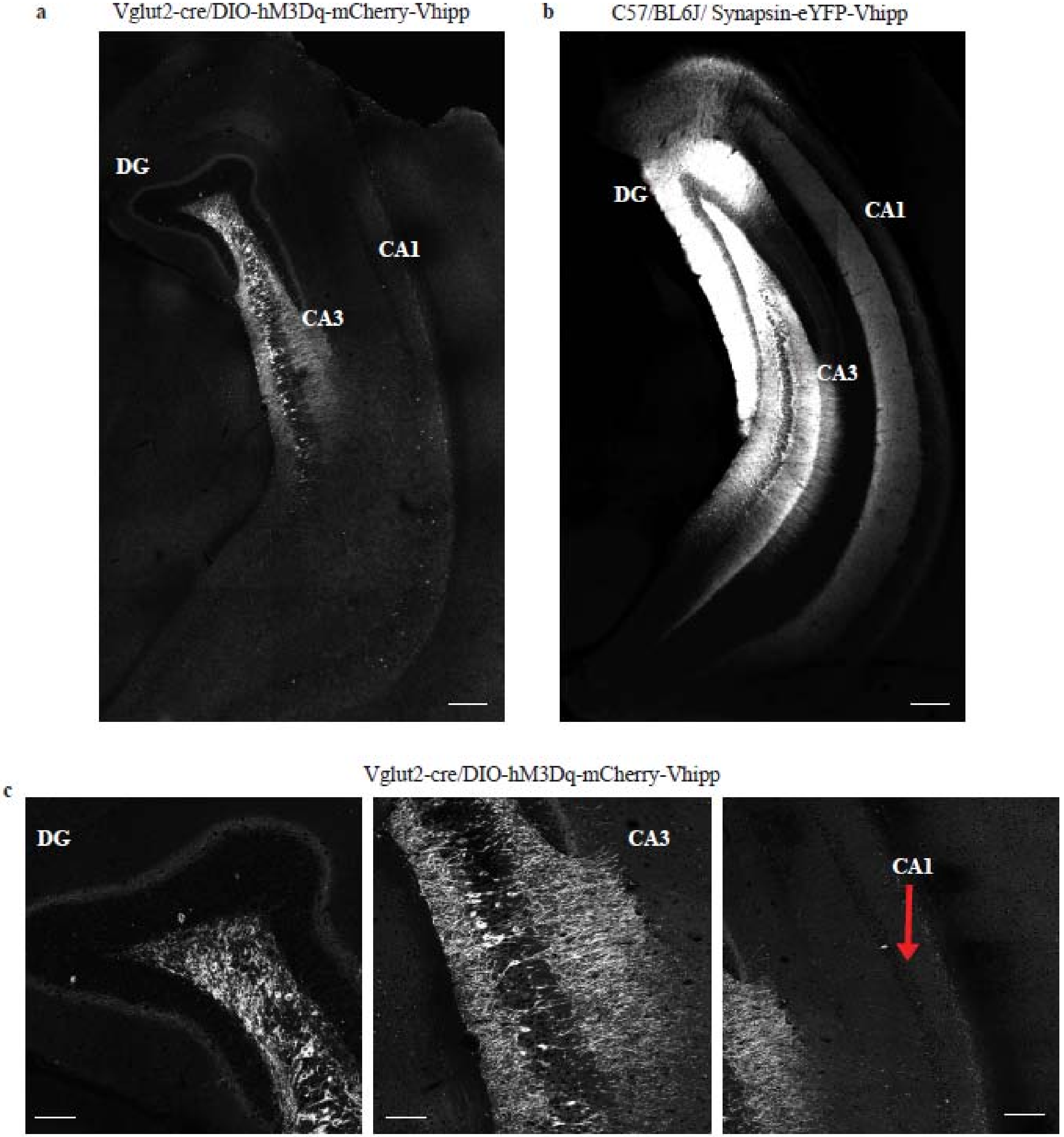

Supplementary Figure 6

Supplemental Figure 6| Comparison of Cre-dependent and Synapsin-driven viral expression in DG and CA3 areas of vHPC (**a**) Representative image showing hM3Dq-mCherry expression in the ventral hippocampus of a Vglut2-Cre mouse. **(b)** Representative image showing Synapsin promoter driven expression of eYFP in the ventral hippocampus of a wild type mouse. Note the stronger expression seen when using the Synapsin promoter when compared to Cre-dependent expression in a Vglut2-Cre mouse. Cre-dependent viral expression is mainly in area CA3 of the ventral hippocampus with sparse labeling observed in CA1. (**c**) Zoomed in images of hM3Dq-mCherry transfection in dentate gyrus (left panel), CA3 (middle panel), and CA1 (right panel). Scale bars, 500 µm for **a and b,** 250 µm for **c** (left panel)**,** 100 µm for **c** (middle and right panel).
